# Fabrication and Characterization of Polycaprolactone/Chitosan—Hydroxyapatite Hybrid Implants for Peripheral Nerve Regeneration

**DOI:** 10.3390/polym13050775

**Published:** 2021-03-03

**Authors:** Katarzyna Nawrotek, Mariusz Mąkiewicz, Dawid Zawadzki

**Affiliations:** Department of Environmental Engineering, Faculty of Process and Environmental Engineering, Lodz University of Technology, Wolczanska 213 Street, 90-924 Lodz, Poland; mariusz.makiewicz@dokt.p.lodz.pl (M.M.); dawid.zawadzki@dokt.p.lodz.pl (D.Z.)

**Keywords:** polymer extrusion, electrophoretic deposition, nerve tissue engineering, hydrogels, implants

## Abstract

Major efforts for the advancement of tubular-shaped implant fabrication focused recently on the development of 3D printing methods that can enable the fabrication of complete devices in a single printing process. However, the main limitation of these solutions is the use of non-biocompatible polymers. Therefore, a new technology for obtaining hybrid implants that employ polymer extrusion and electrophoretic deposition is applied. The fabricated structures are made of two layers: polycaprolactone skeleton and chitosan–hydroxyapatite electrodeposit. Both of them can be functionalized by incorporation of mechanical or biological cues that favor ingrowth, guidance, and correct targeting of axons. The electrodeposition process is conducted at different voltages in order to determine the influence of this process on the structural, chemical, and mechanical properties of implants. In addition, changes in mechanical properties of implants during their incubation in phosphate-buffered solution (pH 7.4) at 37 °C up to 28 days are examined. The presented technology, being low-cost and relatively simple, shall find a broad scope of applications in customized nerve tissue engineering.

## 1. Introduction

Current strategies for reconstruction of damaged peripheral nerves include autografting, allografting, and implantation of nerve guidance conduits (NGCs) to bridge the nerve stumps. Nerve autografting is considered the “gold standard” technique for the repair of peripheral nerve discontinuities. However, this solution has a number of limitations, such as the requirement for a second surgical procedure to harvest the graft tissue, donor site morbidity, additional injuries, scarring, and increased recovery time [1]. Allografts (e.g., cadaver nerve grafts) and xenografts (e.g., animal nerve grafts) can be an alternative to autografts, but their main drawback lies in the high possibility of an undesirable immune response [2]. Therefore, in these cases, immunosuppression therapy, which is costly and has negative impacts on humans, must be applied.

Due to the above-mentioned concerns, the development of novel devices called nerve guidance conduits for peripheral nerve regeneration has received recently a great deal of attention [3]. The most promising materials for the preparation of implants intended for tissue engineering are natural polymers such as collagen, hyaluronic acid, and silk fibroin [4,5,6,7,8]. One of the most widely used synthetic polymers for biomedical applications is polycaprolactone [9].

A promising approach to the peripheral nerve regeneration strategy is to use conduits based on chitosan [10]. The molecular structure of chitosan shows similarity to the glycosaminoglycans of the basal membrane and extracellular matrix (ECM), implicated in cell–cell adhesion [11]. It has been reported that chitosan reveals favorable biological properties including biodegradability, non-toxicity, and remarkable adhesion to living tissues [12]. For the purpose of clinical use, NGCs are required to be easy to fabricate and sterilize and simple to implant in the body by microsurgical techniques. Although chitosan-based materials are proven to be very promising for the fabrication of NGCs [13,14], there is still no efficient and simple method of chitosan processing into tubular forms with appropriate radius and length. Moreover, one of the obstacles in using chitosan materials is their sterilization and aging with time [15]. The storage of materials for biomedical applications often causes changes in their structure, which influences their physicochemical properties. This fact leads to different kinetics of their biodegradation as well as active agent release and, most critically, decomposition of bioactive agents. The other drawback of using chitosan-based materials is connected to their sterilization. Polymer products intended for contact with living tissues must be sterilized before their use. The following common methods of chitosan sterilization can be distinguished: exposure to dry heat, saturated steam, ethylene oxide or radiation. However, before applying any of these methods, their effects on polymer properties and end performance have to be tested, as they can cause irreversible damage to morphological, physical, mechanical, and biological properties. Taking into account the above-mentioned concerns, it seems reasonable to seek a new method of implant production, which will eliminate problems related to the complexity of production and sterilization of chitosan-based materials. In our laboratory, a method allowing us to obtain hydrogel implants within a short time in the process of electrophoretic deposition of chitosan, which eliminates problems related to storage and sterilization of chitosan implants, was developed [16]. Despite its promising potential, there is still a need to incorporate mechanical or biological cues that favor ingrowth, guidance, and correct targeting of axons in a controlled geometry [17,18]. Recently, the potential of 3D printing in tissue engineering applications has been the most studied one. This technique offers many possibilities for fabrication of implants with precisely determined shape, dimensions, mechanical parameters or permeability [19,20]. However, the main disadvantage of 3D printing is the high melting temperature and low degradation rate of commonly applied polymers [21,22]. Most biologically active substances are very sensitive to high temperatures (e.g., temperature of irreversible protein denaturation) and can be easily deactivated [22].

In order to overcome the above-mentioned limitations, a new technology for the production of hybrid implants for the regeneration of peripheral nerves is developed. The proposed strategy employs thermoplastic polymer extrusion and electrophoretic deposition. Polycaprolactone, a polymer characterized by a low melting point, is applied as a filament in the extrusion process. The electrophoretic deposition is conducted using a chitosan–hydroxyapatite solution. The electrodeposition process is conducted at different voltages in order to determine the influence of this process on the structural, chemical, and mechanical properties of implants. The changes in amperage, charge, and gas volume over time are recorded. Fourier-transform infrared spectroscopy (FTIR) and scanning electron microscopy (SEM) are used in order to study implant composition and morphology. In addition, changes in mechanical properties of implants during their incubation in phosphate-buffered solution (pH 7.4) at 37 °C up to 28 days are examined.

## 2. Materials and Methods

### 2.1. Materials

Chitosan (CH, type 85/500) was acquired from Heppe Medical Chitosan GmbH (Halle, Germany). Its degree of deacetylation (DD), viscosity (µ, 1% in 1% acetic acid, 20 °C), and viscosity average molecular weight (Mv) were equal to 82.6–87.5%, 351–750 mPas, and 472 kDa, respectively. Lactic acid (LA), hydroxyapatite (HAp, nanopowder, <200 nm particle size), and polycaprolactone (PCL, average Mn 80,000 mol/wt) were purchased from Merck KGaA (Darmstadt, Germany). Sodium hyaluronate was acquired from Contipro (Dolní Dobrouč, Czech Republic). Its average molecular weight was equal to 2000–2200 kDa.

### 2.2. Apparatus Combining Polymer Extrusion and Electrophoretic Deposition

**Figure 1:**
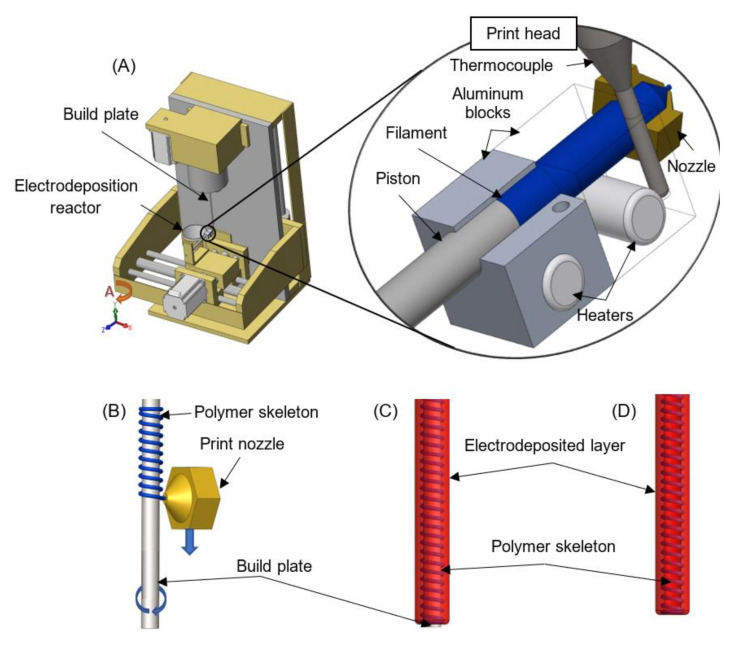
(**A**) Scheme of the apparatus based on polymer extrusion and electrophoretic deposition for fabrication of hybrid implants. Sequence of the manufacturing process: (**B**) polymer extrusion process, (**C**) electrodeposition process, and (**D**) fabricated hybrid implant.

**Figure 2:**
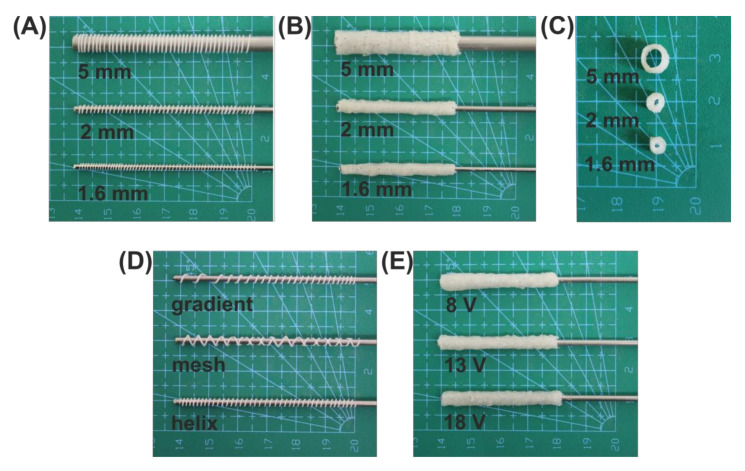
(**A**) Build plate of different diameters with the printed PCL skeleton. (**B**) Longitudinal view and (**C**) transverse cross-section of hybrid implants. (**D**) Exemplary shapes of the PCL skeleton. (**E**) Hybrid implants obtained for the electrodeposition process initiated at different voltages.

A commercially available CNC (Computerized Numerical Control) milling machine with four axes (Shanding U-May Cnc Technology Co., Ltd., Jinan, China) was adapted to work in a vertical position (Figure 1). The apparatus was described earlier in a patent application to the Patent Office of the Republic of Poland: P. 428594 (2019). The device was equipped with a custom-made 3D print head. The head was built of the following elements: two aluminum blocks, two collaterally connected ceramic heaters of a diameter 6 mm (set at 6 W each), a 0.4 mm diameter nozzle, a thermal barrier with an internal polytetrafluoroethylene layer (connecting aluminum blocks and serving as a casing for the melted filament), and a thermocouple coupled with a PID (Proportional–Integral–Derivative) transmitting G8 controller for keeping a constant temperature of 60 °C. The print head was also equipped with a piston. The piston was made of a 2 mm diameter stainless steel rod set in motion by the Z-axis motor of the milling machine. The filament material was placed in a feeder manually. A cylindrical rod made of material consistent with PN-EN ISO 14343 (PN-EN 12072): W 19 9 LSi AWS A5.9: ER 308LSi was used as a build plate. The diameter of the rod can be adjusted to the desired implant dimensions. The presented studies were conducted for rods with diameters of 1.6, 2, or 5 mm (Figure 2). Then, the cylindrical rod was set in motion by the A-axis motor of the milling machine. The CNC milling machine was also equipped with a cylindrical stainless steel reactor for conducing electrophoretic deposition. The inner diameter of the reactor can be adjusted to the desired implant dimensions. The presented studies were conducted for a reactor with an inner diameter of 18 mm.

A G-code used to print polymer skeletons was prepared manually during optimization studies and implemented in Mach3 CNC Controller software (version R3.041, ArtSoft Inc., Livermore Falls, ME, USA). The code was composed of four parts. The aim of the first one was to activate the rotation of the build plate along the A-axis, move the print head from the top to the bottom along the Y-axis, and force out the filament along the Z-axis. This part of the code allows setting parameters responsible for the shape of the polymer skeleton (e.g., helical, mesh or ring structure; constant or gradient pitch). The second part of the G-code was responsible for printing a ring at the end of the polymer skeleton and tearing off the filament thread. The desired effect was obtained through 20 rotations of the A-axis after the set length of the polymer skeleton was obtained. The third part was designed for placing the build plate, which served as an electrode, in the cylindrical reactor for conducing electrophoretic deposition. This included the sequence of the following events: lowering the print head along the Y-axis, positioning the reactor along the X-axis in order to place the build plate in its center, lifting the print head along the Y-axis in order to dip the build plate in solution, and waiting for the set time of electrophoretic deposition. The last part of the G-code was designed for removing the obtained implant from the reactor by lowering the print head along the Y-axis and cleaning the filament chamber by forcing out the residue of the filament.

### 2.3. Implant Fabrication 

Polycaprolactone was applied as a filament for the extrusion of skeletons. Its thermoplastic properties allowed for heating it up to 60 °C. Then, it was formed into a rod of diameter of 1.9 mm and length of 15 mm. Next, the rod was placed into the thermal barrier, reheated to 60 °C, and left at that temperature for 2 min. While the filament was melting, the piston was manually set into the start position. At the same time, the excess of filament was removed by forcing it out. The build plate was placed in a handle and aligned straight along the Y-axis. Then, the reactor for conducting electrophoretic deposition containing the solution was installed. Briefly, the solution was prepared by dissolving 10 mg of hyaluronic acid in 100 mL of 2.5% (*w*/*v*) lactic acid. Then, 1 g of chitosan and 0.09 g of hydroxyapatite were added to the solution. The obtained solution was stirred (under slow rotations) until complete dissolution for 24 h. After dissolution, 16 mL of the solution was poured into the cylindrical reactor with an internal diameter of 20 mm. The next step was to set parameters in the G-code responsible for the shape of the polymer skeleton and the deposition time. After finishing printing of the PCL skeleton, the electrode was moved into the reactor. Then, the electrophoretic deposition process was conducted. After the set time, the obtained hybrid implant was removed automatically from the reactor and taken manually off from the electrode.

### 2.4. Determination of Parameters Influencing the Electrodeposition Process

In order to examine the parameters of the electrodeposition process, the reactor was tightly closed with a cap equipped with an outlet connected to an eudiometer for collecting the evolved gas. The reactor was connected to a power supply and ammeter. Data of amperage were collected by software developed by PC-Link (Link Engine Management Ltd., Christchurch, New Zealand). The electrodeposition process was conducted for 40 min at a constant temperature of 25 ± 0.1 °C and voltage set at 8, 13 or 18 V. The applied time of the process was sufficient for reaching maximum deposition yield. The kinetics of gas emission were tracked using a digital camera and eudiometer. The amount of gas evolved was calculated by applying the ideal gas equation. The mean value of three different measurements was determined, and the standard deviation was calculated for all measurements.

### 2.5. Structural Studies

In order to determine the water content of hydrogels, the deposits were divided into two groups: hydrated and dry. The mass of hydrated specimens was measured just after their preparation. To determine the mass of dry specimens, deposits were placed at 70 ± 0.1 °C for 24 h. The mass of hydrated and dry implants with the PCL skeleton was determined after subtracting the mass of the PCL skeleton. The mean value of three different measurements for both measurements was determined, and the standard deviation was calculated. The water content was determined using the following equation:

(1) $$X=\frac{m_{h}-m_{d}}{m_{h}}\cdot 100\%\;$$

where X—water content (%), m_h_—mass of the deposit (g), and m_d_—mass of the dry deposit (g).

Samples for SEM and FTIR analyses were exposed to a drying procedure. Briefly, hybrid implants were dehydrated for 24 h by placing in an exsiccator containing water and ethyl alcohol (1:1). At the bottom of the exsiccator, calcium chloride was applied. Then, the samples were removed from the exsiccator and left for 24 h to evaporate the ethanol. Dry implants were cut into specimens of length 5 ± 0.2 mm for further examination. Scanning electron microscopy photographs of gold-coated deposits were taken with a Hitachi TM-1000 microscope (Hitachi Ltd., Tokyo, Japan). SEM images were taken as follows: longitudinal view, transverse cross-section, and longitudinal cross-section. FTIR spectra were obtained using a Nicolet™ iS50 FTIR Spectrometer (Thermo Scientific, Madison, WI, USA) equipped with a diamond ATR. The spectra were collected over the range of 500–4000 cm^−1^ for inner and outer surfaces of deposits.

### 2.6. Degradation Studies and Mechanical Testing

The in vitro degradation was performed in 20 mL phosphate-buffered solution (PBS, pH 7.4, Merck KGaA, Germany). Briefly, hybrid implants were incubated in the solution at 37 °C for a period of 1, 7, 14, or 28 days. After the respective times, samples were removed from the medium and subjected to mechanical testing. Mechanical properties of hybrid implants were investigated using an Instron 3345 apparatus (Illinois Tool Works Inc., Norwood, MA, USA). The initial length of specimens was 40 ± 1 mm. The ends of samples were inserted into two 5 mm cylindrical internal supports in order to prevent deformation. Then, the conduit ends were fixed in specially designed clamps. Initially, the distance between the clamps was set to 20 mm. Implants were tested at a strain rate of 3 mm/min at room temperature. Each sample was stretched to complete tensile failure. The mean value of at least three different measurements was determined, and the standard deviation was calculated. After mechanical examination, the samples were dried and weighed.

### 2.7. Statistical Analysis of the Data

One-way analysis of variance (ANOVA) with Tukey’s post-hoc (equal sample sizes) test or the Tukey—Kramer post-hoc (unequal sample sizes) test was used to determine the differences among groups characterized by homogeneity of variance. Welch’s *t*-test with the Games—Howell post-hoc test was used for comparing groups characterized by inhomogeneity of variance. Homogeneity of variance was determined by Levene’s test. The analysis was performed using SPSS Statistics software (version 1.0.0.1447, IBM, New York, NY, USA). *p* < 0.05 was considered statistically significant.

## 3. Results and Discussion

In order to develop a low-cost and relatively simple apparatus for application in customized nerve tissue engineering, a new technology that employs polymer extrusion and electrophoretic deposition is applied (Figure 1). The developed solution allows us to obtain hybrid conduits that structure mimic the chemical composition, mechanical properties, and topography of the extracellular matrix of peripheral nervous tissue. The fabrication of implants is a two-step process. In the first stage, a skeleton is printed on the build plate. In the second step, the electrode is immersed in a solution, and an electric current is applied in order to initiate deposition. The inner diameter of the implant will be dependent on the diameter of the applied build plate (Figure 2). The possibility to use rods with different dimensions is a significant asset for peripheral nerve tissue regeneration, as individual nerve fibers vary widely in diameter within the body.

The role of the inner skeleton is to serve as a scaffolding. The developed G-code allows setting parameters responsible for its shape (Figure 2). The extrusion of skeletons of desired form is realized by the coordination of build plate rotation speed, print head movement, and extrusion speed. The skeleton structure can be printed in the shape of helix, mesh or rings, to name a few. Moreover, the speed of print head movement along the Y-axis can be regulated during the process. Such a solution allows us to obtain a constant or gradient pitch of the printed structure. The filament can be enriched in mechanical or biological cues that favor cell growths, guidance, and correct targeting [23]. In the presented work, polycaprolactone was applied due to its biocompatibility, biodegradability, and wide application in medical fields [24]. Extrusion of the PCL skeleton with the use of the developed apparatus is an effective method. Firstly, the melting point of polycaprolactone is 60 °C. Therefore, it can be easily formed into a filament. When melted, it instantly adheres to the surface of a build plate. At the same time, after solidification, the skeleton is resistant to mechanical factors such as rubbing and hitting. Its surface is characterized by low roughness. Furthermore, during the next step of implant fabrication, electrodeposition, the PCL structure is not damaged by buoyancy and does not undergo rapid hydrolysis. The relatively low melting point of polycaprolactone (compared to other thermoplastic polymers used in 3D printing or polymer extrusion) makes the filament safe for its further biofunctionalization. Most biologically active substances are very sensitive to high temperatures (e.g., temperature of irreversible protein denaturation) and can be easily deactivated [22]. In addition, inclusion of active substances does not need harsh conditions in the developed strategy.

The electrodeposited layer serves as a bonding agent. It is a porous hydrogel, which can be used as a reservoir for biologically active substances responsible for activation and promotion of processes occurring during the recovery of the anatomical connection of axons. The colloidal solution for electrodeposition is prepared by dissolution of chitosan, hydroxyapatite, and sodium hyaluronate in lactic acid water solution. In the acidic environment, amino groups of chitosan are protonated by hydronium ions (H_3_O^+^). Polyelectrolyte chains can interact with water molecules and partially dissolve in water. In the developed apparatus, the electrodeposition process is conducted in the cylindrical stainless steel reactor. The surface of its inner wall serves as an anode, while the build plate covered with the previously extruded PCL skeleton serves as a cathode. Application of an electric current to the described system results in the reduction of water at the surface of the build plate according to the following equation [25]:

(2) $$2H_{2}O+2e^{-}\to 2{\mathrm{OH}}^{-}+H_{2}$$

The above-mentioned reaction initiates alkalization of the closest area of the cathode by increasing hydroxyl ions. Hydrogen is evolved simultaneously. This phenomenon leads to deprotonation of chitosan amino groups according to the following formula:

(3) $$\mathrm{Chit}-NH_{3}^{+}+OH^{-}\to \mathrm{Chit}-NH_{2}+H_{2}O$$

Deprotonated chitosan chains start to form hydrogel deposits on the cathode. However, chitosan is not deposited alone. Co-deposition of hydroxyapatite-derived species is essential in order to obtain hydrogel deposits [26]. The structure of the deposit is irregular and porous due to gas evolution. The ideal material for implants intended for peripheral nerve tissue engineering should be in the form of a membrane allowing the diffusion of oxygen and nutrients into the regeneration site from interstitial fluid. On the other hand, conduit walls should constitute a barrier to the infiltration of inflammatory cells into the conduit lumen. These factors can be controlled by membrane porosity and permeability. The developed method provides full control over pore size and porosity of deposits, mainly by manipulating the initial solution composition and parameters of the electrodeposition process (e.g., time and set voltage) [26]. The deposition process is inhibited after some time due to the presence of hindering factors. The main limitation is the creation of deposits, which hinder hydroxyl ion transport to the deposit–solution interface. As a result, deprotonation of chitosan chains is impossible. The other limitation results from the depletion of substances liable to deposition in colloidal solution. Moreover, a sharp gradient of pH exists only in the proximity of the cathode [25].

The received hybrid implant can be easily removed from the build plate without damaging its structure (Figure 2). Although it is flexible, it is not compressible, i.e., its lumen does not collapse. Such construction is highly desired by surgeons, who can easily put them on resected nerve stamps during surgery [27].

The electrodeposited tubular-shaped implants were described earlier [16,26]. The abovementioned studies focused on the examination of the influence of the PCL skeleton on the process of electrodeposition as well as the chemical composition of implants. In addition, changes in mechanical properties of implants during their incubation in phosphate-buffered solution (pH 7.4) at 37 °C up to 28 days were examined.

### 3.1. Electrodeposition Process Characterization

**Figure 3:**
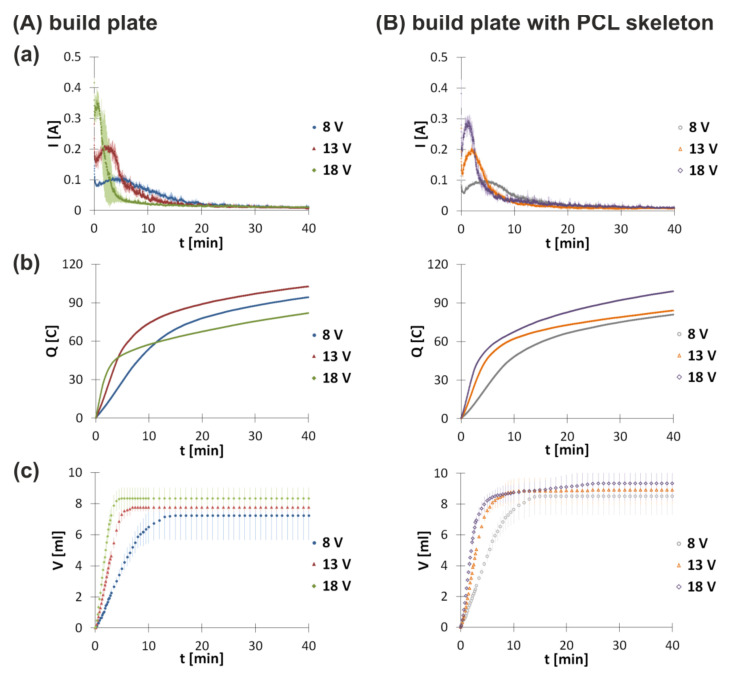
Change in (**a**) amperage, (**b**) charge, and (**c**) gas volume over time for electrodeposition at (**A**) the build plate and (**B**) the build plate with the PCL skeleton initiated at different voltages. Data for amperage and hydrogen volume are shown as the mean ± standard deviation (n = 3).

In order to study the influence of the PCL skeleton on the electrodeposition process, electrodeposition at the build plate without or with the PCL skeleton for 40 min was conducted. The applied time of the process was sufficient for reaching maximum deposition yield. The mass of deposits just after their preparation for electrodeposition run at 8, 13, and 8 V was equal to 0.397 ± 0.017, 0.387 ± 0.025, and 0.363 ± 0.015 g at the build plate and 0.417 ± 0.011, 0.436 ± 0.007, and 0.377 ± 0.034 g at the build plate with the PCL skeleton, respectively. The lower the induced voltage, the higher the water content (Table 1). In addition, deposits obtained at the build plate with the PCL skeleton contained more water than the those obtained at the build plate. The changes in amperage and gas volume are shown in Figure 3. Both deposition at the build plate and deposition at the build plate with the PCL skeleton were characterized by a two-step process. Initially, a rapid amperage drop was observed. This may be caused by the formation of hydrogen bubbles at the surface of the electrode. Their accumulation increases resistance of the colloidal solution. Bubbles detach progressively upon implant thickness growth, which causes co-deposition of chitosan and hydroxyapatite. The forming deposit layer has greater conductibility than hydrogen bubbles. Therefore, amperage slowly increases, reaches a maximum, and decreases. During this period, amperage fluctuations were observed due to continuous hydrogen evolution. The second decrease in amperage after reaching the local maximum results from deposit thickness gain over time. The growing deposit layer is also an obstacle for the diffusion transport of molecules from the solution towards the cathode and vice versa. Both deposition at the build plate and deposition at the build plate with the PCL skeleton are influenced by the set voltage. The higher the voltage, the higher the value of the local amperage maximum observed. Moreover, the local maximum is reached faster for higher voltages. Comparing amperage changes for electrodeposition at the build plate and electrodeposition at the build plate with the PCL skeleton, it can be noticed that the PCL layer does not have a significant influence on the process for voltages 8 and 13 V. For electrodeposition at the build plate at 18 V, the local amperage maximum is equal to 0.357 ± 0.040 A and is reached after 30 s. For electrodeposition at the build plate with the PCL skeleton at 18 V, the local amperage maximum is equal to 0.275 ± 0.011 A and is reached after 83 s.

In order to fully characterize the electrodeposition process at the build plate and electrodeposition at the build plate with the PCL skeleton, the total electric charge exchanged during these processes was calculated (Figure 3b). For this purpose, the definite integral of *I*(*t*) over [0, *t*] was determined from the following equation:

(4) $$Q_{t}=\int_{0}^{t}I\left(t\right)dt\;$$

where *I*—amperage [A] and *t*—time [s].

Both electrodeposition at the build plate and electrodeposition at the build plate with the PCL skeleton are characterized by increasing electric charge over time. At the beginning of the process, a rapid growth of charge is observed as a consequence of higher values of amperage. Moreover, the initial increase in charge is higher for higher voltages for both experiments. Comparing the total electric charge showed some differences for the electrodeposition process at the build plate and electrodeposition at the build plate with the PCL skeleton.

The next parameter measured for electrodeposition both at the build plate and at the build plate with the PCL skeleton is the volume of gas evolved (Figure 3c). For both conditions, similar kinetics are observed. At the beginning, an increase in the volume of evolved gas is noticed. The higher the set voltage, the faster the gas emission. For electrodeposition at the build plate, the gas evolution is inhibited after 15 min at 8 V, 7 min at 13 V, and 5 min at 18 V. The gas emission for the process at the build plate with the PCL skeleton is stopped after 15 min at 8 V, 11 min at 13 V, and 15 min at 18 V. Analyzing results for both conditions, it can be stated that the presence of the PCL skeleton does not have a significant influence on the amount of hydrogen evolved. Upon reduction of water, hydrogen evolution is observed. This process leads to alkalization of the nearest area of the cathode. Deposition and the presence of hydrogen bubbles adhered to the electrode prevent constant particle migration towards the electrode. As a result, deposition as well as hydrogen evolution are hindered.

Table 1 lists values of the total electric charge exchanged (Q_t_) and the total amount of gas collected in the eudiometer (n) for the electrodeposition process at the build plate and at the build plate with the PCL skeleton conducted for 40 min.

The obtained data indicate that the higher the voltage set, the higher the total electric charge exchanged during the process. However, for the experiment at the build plate without the PCL skeleton at 18 V, the total electric charge exchanged is lower than the one for 13 V. The respective values are higher for electrodeposition processes at the build plate with the PCL skeleton compared to electrodeposition processes at the build plate.

### 3.2. Structural Characterization

**Figure 4:**
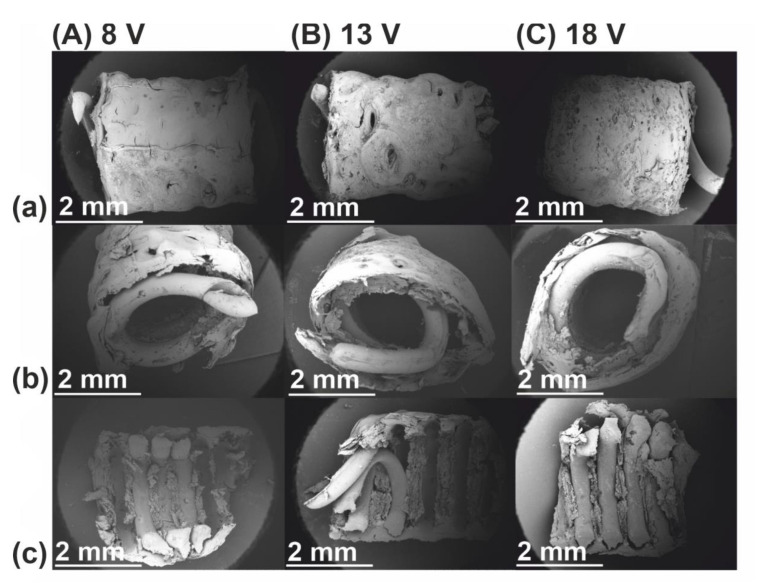
SEM images of 30x magnification of (**a**) longitudinal view, (**b**) transverse cross-section view, and (**c**) inner sidewall view of hybrid implants obtained for the electrodeposition process initiated at (**A**) 8, (**B**) 13, and (**C**) 18 V.

In order to assess the structure as well as incorporation of the PCL skeleton into the electrodeposited layer, scanning electron microscopy was applied (Figure 4). It can be observed that the PCL skeleton does not influence the electrodeposition process and can be entirely covered by the electrodeposit. The easy detachment of the PCL skeleton after drying (Figure 4c) might indicate that it does not create strong molecular interactions with components of the electrodeposited layer. The lower the set voltage, the lower the irregularities of the implant surface. The presence of pores in the bulk of deposits results from hydrogen evolution.

**Figure 5:**
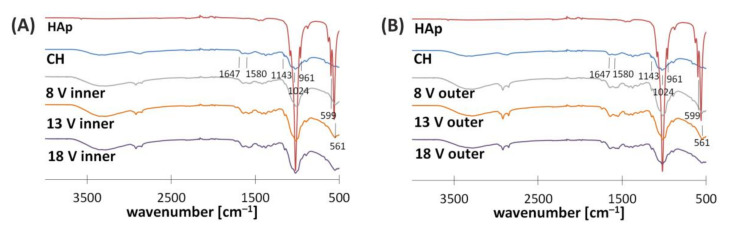
FTIR spectra of (**A**) the inner side and (**B**) outer side of hybrid implants obtained for the electrodeposition process initiated at different voltages.

In order to assess the interaction of compounds forming the electrodeposited layer, FTIR spectroscopy was applied (Figure 5). The obtained spectra are similar for inner and outer surfaces. Furthermore, it seems that structure composition is independent of voltage. For this reason, further discussion treats only the inner side of the implant obtained for the process conducted at 8 V as an example. Peak characteristics for moieties of chitosan are present at: 1647 cm^−1^ (-C=O stretching mode), 1580 cm^−1^ (-NH_2_ bending mode), and three peaks located in the range from 1020 to 1140 cm^−1^ (C-O-C stretching asymmetric and symmetric mode). The spectrum of hydroxyapatite shows peaks associated with the presence of ${\mathrm{PO}}_{4}^{3-}$ at: 1020, 961, 599, and 561 cm^−1^. The stretching mode of the -OH bond is denoted by a peak at 3562 cm^−1^. The spectrum of the inner side of the implant obtained for the process conducted at 8 V shows peaks at 2910 and 2850 cm^−1^. These peaks denote asymmetric and symmetric stretching vibrations of C-H, respectively. Both peaks might result from the presence of chitosan and hyaluronate ions in the electrodeposited layer. The intensity of these peaks is weaker for the inner surface than for the outer one. The peaks indicating the presence of hydroxyapatite-derived moieties are more pronounced for the inner surface. These observations might suggest that the ratio of hydroxyapatite to chitosan–hyaluronate is higher for the inner surface that for the outer one. This can be explained by differences in the mobility of molecules in response to the external electric field. Moreover, deformation of peaks at 1647 and 1580 cm^−1^ suggests the formation of new interactions between chitosan chains and hydroxyapatite-derived moieties. Both the inner and outer surface show weak signals at 1735 cm^−1^, which might indicate the presence of lactate ions [28,29,30,31].

### 3.3. Degradation and Mechanical Properties

**Figure 6:**
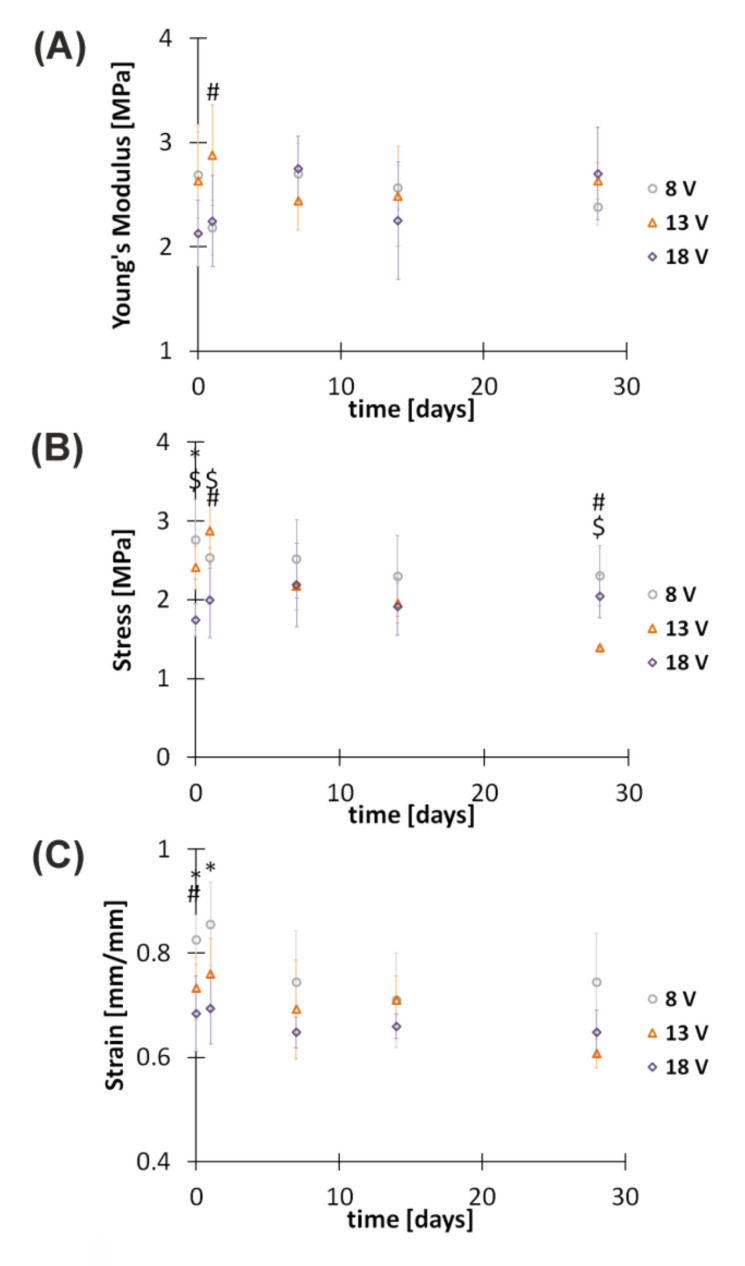
Tensile testing of hybrid implants incubated in PBS (pH 7.4) at 37 °C for specified periods. (**A**) Young’s modulus, (**B**) ultimate stress, and (**C**) ultimate strain. *, #, and $ indicate significant differences between groups of implants obtained in electrodeposition at 8 V and 18 V (*p* < 0.05), 8 V and 13 V (*p* < 0.05), and 13 V and 18 V (*p* < 0.05), respectively.

Mechanical properties were determined by recording Young’s modulus, ultimate stress, and ultimate strain changes for hybrid implants just after preparation and after 1, 7, 14, and 28 days of incubation in PBS solution (Figure 6). Degradation in these conditions is very slow mainly because of pH 7.4, which is higher than the pK_a_ of chitosan (~6.3) [25]. It seems that the main phenomenon responsible for the degradation of hybrid implants is the hydrolysis of glycosidic bonds between polysaccharide rings in chitosan chains [32]. The obtained Young’s modulus, which denotes implant tensile stiffness at breakage, for implants obtained in electrodeposition at 8, 13, and 18 V takes values between 2 and 3 MPa both for implants just after preparation and up to 28 days of incubation in PBS solution. Statistical analysis of Young’s modulus data indicated significant differences (*F*_(2,12)_ *=* 4.56, *p* = 0.034) between groups of implants subjected to electrodeposition at different voltages (Figure 6A). When analyzing ultimate stress, which refers to the force per initial cross-section area of an implant at breakage, it can be observed that the longer the time of incubation, the lower the stress for implants received at 8 and 13 V. The ultimate stress of implants obtained in electrodeposition at 8 V and 13 V is equal to 2.76 ± 0.50 and 2.41 ± 0.27 MPa, respectively. After 28 days of incubation, values decrease to 2.30 ± 0.38 and 1.39 ± 0.03 MPa, respectively. The opposite trend is observed for implants prepared at 18 V. The ultimate stress for these samples increases from 1.74 ± 0.21 to 2.04 ± 0.27 MPa. Statistical analysis of ultimate stress data indicated significant differences (*F*_(2,14)_ *=* 11.23, *p* = 0.001) between groups of implants obtained in electrodeposition at different voltages (Figure 6B). Upon incubation in PBS solution, differences are observed after the following periods: 1 day (*F*_(2,12)_ *=* 6.82, *p* = 0.01) and 28 days (*F*_(2,4.09)_ = 19.40, *p* = 0.009). The next recorded mechanical parameter, ultimate strain, denotes elongation of the implant divided by its original length at breakage. For all studied samples, it takes values between 0.6 and 0.9 mm/mm both for implants just after preparation and up to 28 days of incubation in PBS solution. Statistical analysis of ultimate strain data indicated significant differences (*F*_(2,14)_ = 8.11, *p* = 0.0046) between groups of implants obtained in electrodeposition at different voltages (Figure 6C). Upon incubation in PBS solution, differences are observed after 1 day (*F*_(2,12)_ = 6.26, *p* = 0.013). Small changes in implant mechanical properties during their incubation in the physiological environment over the time needed for axon outgrowth (approximately 28 days) are desired. The lack of significant changes in Young’s modulus, ultimate stress, and ultimate strain indicates the presence of hydrophobic interactions between the implant components and incubation solution.

**Figure 7:**
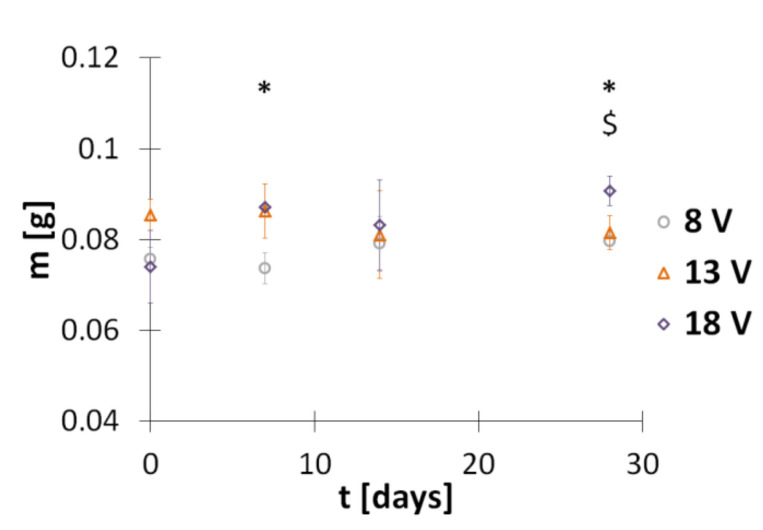
Mass of hybrid implants incubated in PBS (pH 7.4) at 37 °C for specified periods. * and $ indicate significant differences between groups of implants obtained in electrodeposition at 8 V and 18 V (*p* < 0.05), and 13 V and 18 V (*p* < 0.05), respectively.

Another parameter tracked in order to assess the degradation of hybrid implants in PBS water solution was the mass of dried conduits over time. The obtained results are shown in Figure 7. The mass of dry implants obtained in electrodeposition at 8, 13, and 18 V is equal to 0.1258 ± 0.0035, 0.1352 ± 0.0028, and 0.1271 ± 0.0091 g, respectively. It seems that the voltage influences the mass of the implant. Statistical analysis of mass data indicated significant differences between groups of implants obtained in electrodeposition at different voltages after their incubation in PBS solution for 7 days (*F*_(2,3.61)_ = 7.24, *p* = 0.01) and 28 days (*F*_(2,8)_ = 12.68, *p* = 0.007) (Figure 7). The data in Figure 7 show that even very long timesof incubation in PBS solution does not change the implant mass.

In analyzing requirements for implants intended for peripheral nerve tissue engineering, the appropriate mechanical properties should be referred to those of peripheral nerve tissue. For example, Young’s modulus, ultimate stress, and ultimate strain of fresh rat sciatic nerves are equal to 0.580 ± 0.150 MPa, 2.720 ± 0.97 MPa, and 0.810 ± 0.114 mm/mm, respectively [33]. Based on the obtained results, it can be concluded that the mechanical properties of the developed hybrid implants are sufficient to fulfil the requirements of peripheral nerve tissue engineering.

## 4. Conclusions

A novel technology that employs polymer extrusion and electrophoretic deposition intended for production of hybrid implants is described. The obtained results indicate that the developed strategy is an effective method for incorporation of a PCL skeleton within chitosan–hydroxyapatite hydrogel deposit. The possibility to change the shape of the extruded PCL structure is a valuable tool for incorporation of mechanical or biological cues that favor cell ingrowth, guidance, and correct targeting of axons. The water content in the hydrogel deposit is dependent on voltage. Therefore, the rate of diffusion of oxygen and nutrients into the regeneration site from interstitial fluid can be controlled. The mechanical properties of implants during incubation in phosphate-buffered solution (pH 7.4) do not change significantly up to 28 days at 37 °C. As the developed automatic apparatus is designed to produce implants in a short time, the developed technology can be regarded as more effective compared to existing solutions. The next studies will be focused on application of the received implants for appropriate cellular and animal models.

## 5. Patents

Patent application sent to the Patent Office of the Republic of Poland: P. 428594 (2019).

## Figures and Tables

**Table 1:** Quantitative characteristics of electrodeposition processes at the build plate and at the build plate with the PCL skeleton. Q_t_—total electric charge, n—amount of evolved gas, and X—water content.

Electrodeposition at the Build Plate			
U [V]	8	13	18
Q_t_ [C]	94.4	102	81.9
n [μmol]	294	316	340
X [%]	75.9	76.4	73.7
**Electrodeposition at the Build Plate with the PCL Skeleton**			
Q_t_ [C]	80.9	84.1	99.0
n [μmol]	361	360	377
X [%]	77.7	76.2	74.7

## Data Availability

The data presented in this study are available on request from the corresponding author.
